# Bio-Engineered Scaffolds Derived from Decellularized Human Esophagus for Functional Organ Reconstruction

**DOI:** 10.3390/cells11192945

**Published:** 2022-09-20

**Authors:** Silvia Barbon, Andrea Biccari, Elena Stocco, Giovanni Capovilla, Edoardo D’Angelo, Martina Todesco, Deborah Sandrin, Andrea Bagno, Filippo Romanato, Veronica Macchi, Raffaele De Caro, Marco Agostini, Stefano Merigliano, Michele Valmasoni, Andrea Porzionato

**Affiliations:** 1 Section of Human Anatomy, Department of Neuroscience, University of Padova, 35121 Padova, Italy; 2 L.i.f.e.L.a.b. Program, Consorzio per la Ricerca Sanitaria, 35128 Padova, Italy; 3 Foundation for Biology and Regenerative Medicine, Tissue Engineering and Signaling—TES, Onlus, 35136 Padova, Italy; 4 Department of Surgical Oncological and Gastroenterological Sciences, University of Padova, 35128 Padova, Italy; 5 Department of Industrial Engineering, University of Padova, 35131 Padova, Italy; 6 Department of Physics and Astronomy “G. Galilei”, University of Padova, 35131 Padova, Italy

**Keywords:** human esophagus, decellularization, extracellular matrix, allograft, tissue engineering

## Abstract

Esophageal reconstruction through bio-engineered allografts that highly resemble the peculiar properties of the tissue extracellular matrix (ECM) is a prospective strategy to overcome the limitations of current surgical approaches. In this work, human esophagus was decellularized for the first time in the literature by comparing three detergent-enzymatic protocols. After decellularization, residual DNA quantification and histological analyses showed that all protocols efficiently removed cells, DNA (<50 ng/mg of tissue) and muscle fibers, preserving collagen/elastin components. The glycosaminoglycan fraction was maintained (70–98%) in the decellularized versus native tissues, while immunohistochemistry showed unchanged expression of specific ECM markers (collagen IV, laminin). The proteomic signature of acellular esophagi corroborated the retention of structural collagens, basement membrane and matrix–cell interaction proteins. Conversely, decellularization led to the loss of HLA-DR expression, producing non-immunogenic allografts. According to hydroxyproline quantification, matrix collagen was preserved (2–6 µg/mg of tissue) after decellularization, while Second-Harmonic Generation imaging highlighted a decrease in collagen intensity. Based on uniaxial tensile tests, decellularization affected tissue stiffness, but sample integrity/manipulability was still maintained. Finally, the cytotoxicity test revealed that no harmful remnants/contaminants were present on acellular esophageal matrices, suggesting allograft biosafety. Despite the different outcomes showed by the three decellularization methods (regarding, for example, tissue manipulability, DNA removal, and glycosaminoglycans/hydroxyproline contents) the ultimate validation should be provided by future repopulation tests and in vivo orthotopic implant of esophageal scaffolds.

## 1. Introduction

Gastric or colonic transpositions and, more rarely, jejunal grafts constitute the mainstay of reconstructive treatment after esophageal resection for benign disorders (including caustic burns and atresia) or cancer. However, these reconstructions are burdened by a high rate of postoperative complications and mortality and, in the long term, a reduced quality of life due to reflux, delayed emptying, dumping syndrome and strictures. Moreover, in particularly complicated cases, after the failure of the reconstructive techniques or unsuitability of the organs for reconstruction, clinicians could face a therapeutic dead-end where no reconstructive strategies are available, and patients require life-long enteral feeding through jejunostomy. In this context, the development of tissue-engineered (TE) substitutes, allowing esophageal replacement to be performed without sacrificing other organs, is desirable.

The use of decellularized scaffolds might be the best option to achieve this goal, as decellularized extracellular matrix (ECM) can preserve organs’ architecture and guide cellular migration, anchorage and 3D organization [1,2,3,4,5]. In recent years, the development of tissue-specific decellularized matrices has rapidly expanded and successful clinical applications have been achieved to regenerate children’s airways [6], for cardiac valves replacement [7] and human dermal reconstruction [8].

The esophagus poses unique challenges in the successful creation of a TE graft, as it is multi-layered and has a complex vascular network and innervation [9]; therefore, the transplantation of appropriate cell linings is crucial to achieve the regeneration of all functional compartments [10,11].

At present, several TE esophageal substitutes realized using decellularized ECM have been successfully used in clinical practice as patches to repair small defects [12,13,14] or to repair mucosal lesions [15,16]. The achievement of full-thickness circumferential grafting is currently substantially limited by inflammation, fibrosis and stricture formation [12,13,14,17].

Circumferential decellularized-ECM scaffolds have been developed in small animal and porcine models [5,10,18]; however, to the best of our knowledge, no data are available on the development of an ECM scaffold from the decellularization of a human esophagus from cadaveric donors. We hypothesized that a human-derived esophageal scaffold could retain the complex extracellular matrix components, and the capacity to modulate recellularizations, while reducing immunogenicity and thus related inflammation [19,20,21]. The harvesting of esophageal tissue for future clinical use could be integrated in existing organ donation programs. Based on these considerations, the aim of this work was to test and compare three detergent-enzymatic methods for the decellularization of human esophagus. Specifically, selected protocols were chosen based on previous experience of our research group on the successful decellularization of gastroenteric (i.e., colorectal) [22] and muscular [23,24] tissues. After decellularization, the quality of the resulting acellular matrix was assessed via the immunohistological, biochemical, proteomic and biomechanical characterization of scaffold structure and composition.

## 2. Materials and Methods

Reagents and consumables were mainly purchased from Merck Life Science (Darmstadt, Germany), unless otherwise specified.

### 2.1. Collection and Sampling of Human Esophagus

Human esophageal samples were collected from cadavers, provided by Body Donation Program of the Section of Human Anatomy, University of Padova [25,26,27,28], according to European, Italian and regional guidelines [29,30]. After esophagus excision from the cervical to the middle thoracic portion, the sample was cleaned from the surrounding tissue debris (i.e., adipose tissue). Esophagus was then dissected to obtain 2.5 cm tubular segments or 2.5 × 2.5 cm^2^ patches, and specimens were stored at −80 °C until further processing. At the moment of esophagus sampling, the control specimens of native tissue were fixed with 10% formalin or snap-frozen at −80 °C for subsequent analyses.

### 2.2. Tissue Decellularization

In order to avoid contamination by microorganisms, esophageal samples were manipulated under sterile conditions during all the procedures, and all decellularization solutions were sterile-filtered. Tubular segments and patches were thawed at room temperature (RT) and further cleaned from surrounding tissue debris before performing extensive washes with 3% antibiotic solution in phosphate-buffered saline (PBS), in order to remove any contaminants. Progressive washes were then carried out with antibiotic solution in decreasing concentrations, with the last wash in PBS only, to eliminate any residual antibiotic.

Three detergent-enzymatic protocols were tested for human esophagus decellularization, as detailed in Table 1. Esophageal tubules (ETs) decellularized with Protocols Nos. 1 [22], 2 and 3 were named ET1, ET2 and ET3, respectively. Similarly, esophageal patches (EPs) decellularized with Protocols Nos. 1, 2 and 3 were named EP1, EP2 and EP3, respectively. As controls, non-treated samples named ETN (esophageal tubule, native) and EPN (esophageal patch, native) were considered. Protocol No. 1 was repeated for 2 cycles to obtain ET1 and for 3 cycles to obtain EP1.

The experimental procedures for each protocol are briefly described below.

*Protocol No. 1:* Each detergent-enzymatic treatment (DET) cycle consisted of a first wash in deionized water (dH_2_O) at 4 °C for 24 h, followed by sample treatment with 4% sodium deoxycholate (SDC) at RT for 4 h and with 2000 kU DNase-I in 1 M sodium chloride (NaCl) at RT for 3 h. These steps were performed under agitation, at a speed of 100 oscillations/minute. Then, tissues were washed in dH_2_O overnight. After decellularization, matrices were rinsed in 3 % penicillin/streptomycin (pen/strep)/PBS for at least 3 days. Finally, samples were sterilized in peracetic acid at 0.1 M for 1 h at RT under agitation and preserved at −80 °C until use.

*Protocols Nos. 2*, *3:* Esophageal samples were washed with dH_2_O for 24 h at 4 °C, incubated for 1 h with 0.05% trypsin-0.02% EDTA at 37 °C and then treated with 0.002% SDS (Protocol No. 2) or 2% Tergitol^TM^ (Protocol No. 3) + 0.8% ammonium hydroxide (NH_4_OH) for 72 h at 4 °C under continuous agitation (speed: 100 oscillations/minute). Finally, samples were washed with dH_2_O for 72 h and preserved at –80 °C until use.

After the detergent-enzymatic treatments, the appearances of the decellularized and native esophagi were compared by assessing tubule/patch weight, whereas tissue integrity was evaluated by measuring the main dimensions of samples; the length, as the largest horizontal dimension, and the width of esophageal specimens were calculated using macroscopic images elaborated with Fiji software (NIH, Bethesda, MD, USA).

### 2.3. DNA Isolation and Quantification 

To assess total DNA content in the native samples compared with decellularized matrices, 20 mg of each specimen was treated using a DNeasyBlood&Tissue kit (Qiagen, Hilden, Germany) according to the manufacturer’s instructions. DNA samples were then quantified using Nanodrop 2000 at the 260/280 nm ratio (ThermoFisher Scientific, Waltham, MA, USA).

### 2.4. Histological Investigations

After the decellularization process, treated samples were fixed with 10% formalin, paraffin-embedded, cut into 5 μm thick sections and investigated using histological analyses according to routine protocols in order to verify the effective removal of cell nuclei and the maintenance of ECM structure and composition in comparison with native cellular tissue. Before staining, sections were de-waxed and rehydrated with a series of ethanol (Arco Scientifica S.r.l., Padua, Italy) solutions (99%, 95%, 70%) and distilled water. Subsequently, hematoxylin and eosin (H&E) staining was performed to verify the effective removal of cell nuclei and muscle fibers, and Weigert Van Gieson staining was carried out to demonstrate the persistence of elastic fibers, while Masson’s trichrome staining detected preserved collagen, confirming the absence of muscle fibers.

### 2.5. Immunohistochemical Study

The immunolocalization of Collagen IV and Laminin within the decellularized samples was performed to assess the adequate preservation of tissue-specific ECM proteins in comparison with the native control. At the same time, the non-immunogenicity of the acellular scaffolds was verified via the investigation of MHC class II (HLA-DR) antigens. Immunohistochemical reactions were carried out using Dako Autostainer/Autostainer Plus (Dako, Milan, Italy) with the following antibodies diluted in PBS: anti-Collagen IV (monoclonal mouse anti-COL4A3; sc-52317; Santa Cruz Biotechnology, Dallas, TX, USA) (1:100); anti-Laminin (polyclonal rabbit anti-LAM; L9393; Merck Life Science) (1:200); and anti-HLA-DR (monoclonal mouse anti-HLA-DR antigens; M0746; Dako) (1:50). Except for Laminin, epitope retrieval was performed with 10 mM sodium citrate buffer, pH 6.0 (for HLA-DR) or pH 9.0 (for Collagen IV), at 90 °C for 10 min (min). Sections were then incubated with peroxidase-blocking serum (EnVision FLEX Peroxidase-Blocking Reagent; Dako) for 5 min in order to avoid unspecific binding before incubation for 1 h at RT with the above primary antibodies. The specific binding of the primary antibodies was revealed by means of incubation with the secondary antibodies (EnVision FLEX Mouse-Linker and EnVision FLEX Rabbit-Linker; Dako) for 15 min and EnVision FLEX/HRP polymer for 20 min. Subsequently, 3,3′-diaminobenzidine (EnVision FLEX Substrate Buffer + DAB + Chromogen; Dako) was used in order to highlight the positivity of the reaction. Finally, the sections were counterstained with hematoxylin. Native diaphragmatic samples were used as reference for marker expression, whereas negative controls were prepared by incubating sections without primary antibodies.

### 2.6. ECM Component Quantification

Sulphated glycosaminoglycan (GAG) content into native and decellularized esophageal tissues (10 mg) were quantified by using Chondrex Inc. Glycosaminoglycans Assay Kit (DBA Italia S.r.l, Milan, Italy) according to the manufacturer’s instructions. In parallel, to quantify the collagen component in native and decellularized tissues (5 mg), a Hydroxyproline assay kit was used following the manufacturer’s instructions.

### 2.7. Scanning Electron Microscopy (SEM)

Samples were fixed with 2% glutaraldehyde in 0.1 M phosphate; following washing with deionized water, they were cut into segments of approximately 1 cm in length, cryoprotected in 25% sucrose and 10% glycerol in 0.05 MPBS (pH 7.4) for 2 h and then fast frozen. At the time of analysis, samples were placed back into the cryoprotectant at RT and allowed to thaw. After washing, the material was fixed in 1% OsO_4_/0.1 M phosphate buffer (pH 7.3) and washed again. After rinsing with deionized water, specimens were dehydrated in a graded ethanol–water series to 100% ethanol, critical-point-dried using CO_2_ and finally mounted on aluminum stubs using sticky carbon taps. Samples were mounted and coated with a thin layer of Au/Pd (approximately 2 nm thick) using a Gatan ion beam coater. Images were recorded with a Jeol 7401 FEG scanning electron microscope (Akishima, Tokyo, Japan).

### 2.8. Second-Harmonic Generation Microscopy

Second-Harmonic Generation (SHG) imaging was performed on decellularized esophageal tubules and patches in comparison with the native tissues using a custom developed multiphoton microscope, previously described by Filippi et al. [31]. In brief, an incident wavelength of 800 nm was adopted to detect the collagen’s SHG signal at 400 nm and the AutoF signal at 525 nm on two different photodetectors (GaAsP PMT with a 395/25 nm bandpass filter and GaAsP PMT with a 525/40 nm bandpass filter, respectively). The images were acquired at a fixed magnification through the Olympus 25× water immersion objective with 1.05 numerical aperture (1024 × 1024 pixels), averaged over 70 consecutive frames, with a pixel dwell time of 0.14 μs and a pixel width of 0.8 μm. For quantitative measurements, the RAW uncompressed images were analyzed using Image-J software, version 1.8.0. Coherency (C) was calculated for collagen and elastin to verify the local dominant orientation of the images using OrientationJ, an ImageJ plugin [32]. The parameter is bounded between 0 and 1, indicating the absence (isotropy) and the presence (anisotropy) of dominant orientation, respectively. A graphic representation of coherency that shows the organization and distribution of the fibers is given via Fast Fourier Transform (FFT) analysis. The transform-based texture analysis techniques convert the image into a new form using the spatial frequency properties of the pixel intensity variations, allowing one to extract textural characteristics from the image. Indeed, highly oriented fiber in a single direction shows an elliptic shape; differently, a circular shape represents fiber spread in all directions [33,34].

### 2.9. Mass Spectrometry Analysis

#### 2.9.1. Sample Preparation

Decellularized esophageal matrices (range of 2–5 mg) were digested following the protocol proposed by Naba et al. [35]. Briefly, samples were reduced, alkylated, deglycosylated (PNGaseF; 500,000 U/mL; New England Biolabs, Ipswich, MA, USA) and digested with Lys-C (0.1 μg/μL; New England Biolabs) and Trypsin (0.5–0.1 μg/μL; Sequencing Grade Modified Tyrpsin, PROMEGA Italia Srl, Milan, Italy) enzymes. After digestion, samples were purified with SPE (Supel^TM^-Select HLB SPE tubes, Supelco – Merck LifeScience). Eluted peptides were finally dried under vacuum and stored at −20 °C until analysis.

#### 2.9.2. Protein Identification

Samples were resuspended in different volumes of 5% CH_3_CN + 0.01% formic acid solution to obtain equal column loading and were analyzed using a UHPLC-XEVO-G2-XS (Waters, Milford, MA, USA) mass spectrometer. Peptides were separated with a Biobasic (Milan, Italy) C18 column, 150 mm × 1 mm ID, 5 μm, using a 3–45% linear gradient of CH_3_CN + 0.1% TFA (mobile phase B) in H_2_O + 0.1% TFA (mobile phase A) over 110 min. Mass spec data were acquired in data-dependent mode in the 350–2000 m/z mass range. The instrumental parameters were set as follows: source, ESI (+); precursor charge selection, from 2 to 4; resolution, 22,000 FWHM. 

#### 2.9.3. Data Processing

Mass spec data were lock-mass corrected, peak picked, converted into mzML format using Trans Proteomic Pipeline (TPP) and processed with Proteome Discoverer 2.2 (ThermoFisher Scientific). The search parameters were set as follows: database, UP000005640 UniProt reference proteome: enzyme, Trypsin (max 2 missed cleavages); taxonomy, *homo sapiens*; precursor mass tolerance, 25 ppm, fragment mass tolerance, 0.08 Da. Fixed modifications: carbamidomethyl (C). Dynamic modifications: oxidation (M, P, K); deamidation (N, Q), and phosphorylation (S, T, Y). A minimum of 2 non-redundant peptides were used for protein identification.

### 2.10. Mechanical Properties 

The mechanical properties of native and decellularized esophageal samples were evaluated with a uniaxial tensile test. Due to limited material availability, tests were performed on native and decellularized human esophagus patches, not on tubules. Esophageal patches were cut into dog-bone-shaped specimens with a gauge length of 5 mm and a 2 mm width, according to the ASTM D1708-13 standard concerning small-size tissues [36]. This allowed us to analyze sample response to the tensile force applied in both the longitudinal (along the main axis) and circumferential (perpendicular to the main axis) directions. Native and decellularized esophagi underwent uniaxial tensile loading tests using a custom-made apparatus (IRS, Padova, Italy) equipped with four linear actuators and four loading cells (50 N). Uniaxial tests were performed using two actuators and two cells at RT and by continuously hydrating samples with 0.9 % sodium chloride (NaCl) solution. Samples were first preloaded up to 0.1 N and then elongated (elongation rate of 0.2 mm/s) up to 300% of the initial length, in order to reach tissue rupture for measuring the Ultimate Tensile Strength (UTS) and the Failure Strain (FS). Stress–strain curves were obtained for each specimen, where (a) engineering stress σ (MPa) was defined as the tensile force measured by the loading cells (Newton) divided by the original cross-sectional area of the sample, and (b) strain ε (%) was defined as the ratio between the grip displacement and the gauge length. Elastic modules E_1_ and E_2_ were calculated as the slope of the stress–strain curves between 1 and 10% of deformation and between 80 and 90% of deformation, respectively. Finally, the toughness (I) was defined as the energy required (per unit of volume) to bring the material to failure.

### 2.11. Cytotoxicity Study

#### 2.11.1. HM1SV40 Cell Cultures

The immortalized human bone marrow cell line HM1-SV40 [37] was cultured in proliferation medium containing Alfa-Modified Eagle Medium (α-MEM) Without Nucleosides (ThermoFisher Scientific), 16.5% fetal bovine serum (FBS) (ThermoFisher Scientific), 1% glutamine and 1% penicillin/streptomycin solution (100 mg/mL). Cells were kept at 37 °C, 5% CO_2_ and 95% humidity for 3–4 days for in vitro expansion; then, they were seeded at a density of 10,000 cells/cm^2^ into 24-well plates and let adhere for 24 h before performing the cytotoxicity test.

#### 2.11.2. Preparation of Conditioned Culture Media

The release of any leachable remnants by esophageal matrices after the decellularization treatments was verified with the cytotoxicity extract test [24,38]. For Protocols n. Nos. 2 and 3, which did not include a final sterilization step, decellularized esophagi were sterilized with 2% antibiotic/antimycotic solution in PBS for 72 h at RT under agitation (100 oscillations/minute) and subsequent extensive washing in PBS for another 72 h.

Esophageal tissues decellularized through Protocol No. 1, No. 2 and No. 3 were then incubated in HM1-SV40 cell proliferation medium (100 milligrams of tissue per milliliter) for 72 h at 37 °C, 5% CO_2_ and 95% humidity. Conditioned media were then used to culture HM1-SV40 cells seeded on 24-well plates as previously described.

#### 2.11.3. Cell Viability Assay

The cytocompatibility of acellular esophageal matrices was assessed by treating HM1-SV40 cells with conditioned media for 72 h. In parallel, untreated cultures and cells incubated under cytotoxic conditions (50% Dimethyl sulfoxide, DMSO) were considered as negative and positive controls, respectively. At the end of the 72 h incubation period, cell viability, proliferation and cytotoxicity under different culture conditions were assessed using the MTT assay, which measures cellular metabolic activity. In brief, cells were incubated for 4 h in basal medium (α-MEM) supplemented with 0.5 mg/mL (3-(4,5-dimethylthiazol-2-yl)-2,5-diphenyltetrazolium bromide (MTT). After that, the incubation buffer was removed, and the violet MTT-formazan product was extracted with acidified isopropyl alcohol (0.04 M HCl in isopropyl alcohol) (Carlo Erba, Milan, Italy). After 15 min of extraction at RT under gentle stirring, the optical density of the formazan solutions was read at 570 nm by analyzing samples with a VICTOR3™ Microplate auto reader (PerkinElmer, Waltham, MA, USA). Results were expressed as percentage of cell viability in treated samples versus the untreated negative control (100% viability).

### 2.12. Statistical Analysis

All graphs and statistical analysis were performed using GraphPad Prism Software 7. Data were expressed as means ± SEMs. For the comparison of paired experimental groups, two-sided Student’s *t*-tests (for the parametric dataset) and Mann–Whitney test (for the non-parametric dataset) were used. One-way ANOVA with Bonferroni’s post hoc test (for the parametric dataset) and Kruskal–Wallis test with Dunn’s post hoc test (for the non-parametric dataset) were performed for multiple comparisons. A *p*-value < 0.05 was considered statistically significant (* *p* < 0.05, ** *p* < 0.01, *** *p* < 0.001, **** *p* < 0.0001). 

## 3. Results

### 3.1. Decellularization of Human Esophagus

After decellularization, all the esophageal samples seemed to preserve a certain volume and integrity in comparison with the native tissues (Table 2). No tissue ruptures or damage were observed following the detergent-enzymatic treatments. The esophageal tubules remained patent and retained the overall shape of the native esophagus.

**Figure 1:**
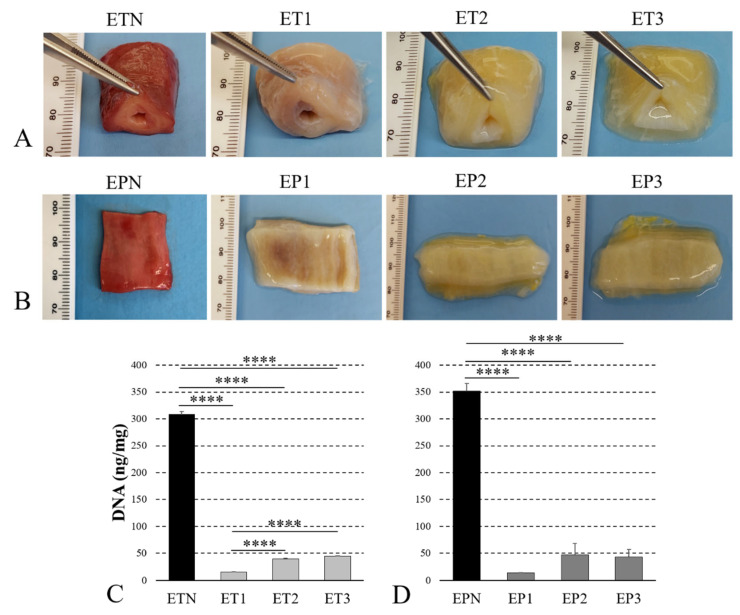
Decellularization efficacy. Gross appearance (**A**,**B**) of esophageal tubules (ETs) and patches (EPs) before (ETN, EPN) and after decellularization with Protocols Nos. 1 (ET1, EP1), 2 (ET2, EP2) and 3 (ET3, EP3). Quantification of residual DNA (**C**,**D**) into decellularized versus native samples (**** *p* < 0.0001).

According to preliminary macroscopic evaluation, the treated tubules and patches turned translucent and white in comparison with the native specimens, likely due to the loss of the cellular and myofibrillar components (Figure 1A,B). In particular, samples ET1 and EP1 appeared to retain a more pinkish color of the muscle layer, suggesting some persistence of myofibrils. On the other hand, samples ET2, ET3, EP2 and EP3 showed a white color in both mucosal and muscular layers. As expected, the tissue consistency of both tubular samples and patches seemed to decrease after decellularization in comparison with the native control; however, all samples maintained good manipulability, except for ET3 and EP3, which tended to collapse during handling.

The quantification of residual immunogenic material showed a significant decrease in the DNA content into all the decellularized scaffolds in comparison with the native counterparts, getting under the threshold of 50 ng/mg of tissue previously indicated to consider efficient sample decellularization [39] (Figure 1C,D). In both tubules and patches, Protocol No. 1 led to a significantly lower level of residual DNA in comparison with Protocols Nos. 2 and 3.

### 3.2. Histological Evaluation

**Figure 2:**
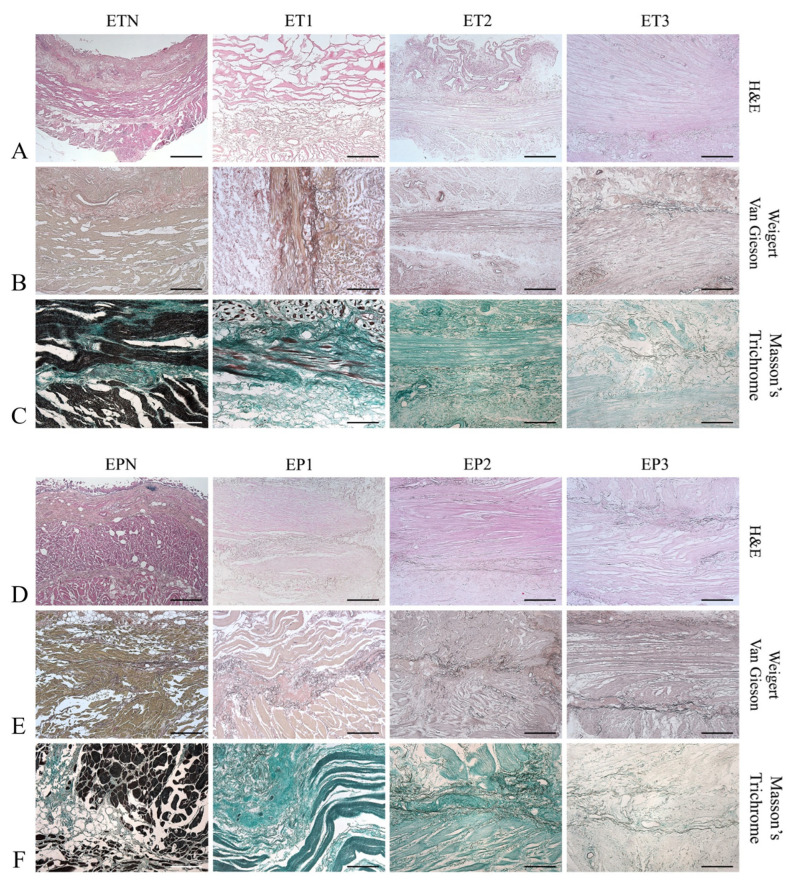
Microscopic structure of decellularized esophagi. Histological evaluation of esophageal tubules (ETs) and patches (EPs) before (ETN, EPN) and after decellularization with Protocols Nos. 1 (ET1, EP1), 2 (ET2, EP2) and 3 (ET3, EP3). Scale bars: 800 µm ((**A**,**D**) ETN, EPN); 400 µm (**A**,**B**,**D**,**E**); 200 µm (**C**,**F**).

The efficient removal of cells and the maintenance of the native structure was assessed using H&E staining, which showed the absence of violet nuclear content in all decellularized esophageal samples, as well as the preservation of the pink eosinophilic staining typical of collagen in acellular scaffolds. Muscle fibers were correctly removed via all protocols, although ET1 and EP1 samples showed a certain persistence of myofibrils within some areas of the tissue slices (Figure 2A,D).

Overall, the general architecture was maintained after decellularization, and the different layers of the esophageal matrix could be distinguished. Weigert van Gieson staining highlighted the persistence of black-colored elastic fibers in the lamina propria, between the muscularis mucosa and the muscularis externa, and within the wall of the blood vessels (Figure 2B,E). Masson’s Trichrome staining revealed the maintenance of green-colored collagen fibers in the acellular esophageal ECM, with a much weaker color observed for samples ET3 and EP3 (Figure 2C,F).

### 3.3. Immunohistochemical Study

**Figure 3:**
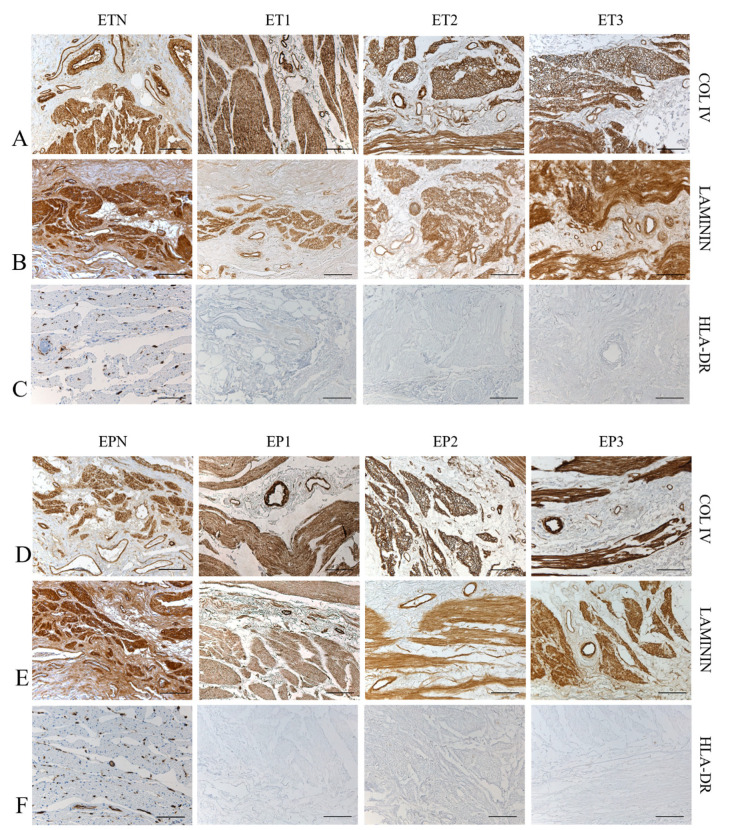
Preservation of tissue-specific markers. Immunohistochemistry for the localization of specific ECM markers and MHC II antigens within esophageal tubules (ETs) and patches (EPs) before (ETN, EPN) and after decellularization with Protocols Nos. 1 (ET1, EP1), 2 (ET2, EP2) and 3 (ET3, EP3). Scale bar: 100 µm (**A**–**F**).

Besides histological evaluation, immunohistochemistry confirmed the retention of different ECM-specific markers in decellularized tissue scaffolds (Figure 3). The expression of laminin was detected in the submucosa and lamina propria, as well as in the blood-vessel wall (Figure 3B,E).

Additionally, Collagen type IV was mainly localized in the muscular layers, surrounding muscle fibers, and around the blood vessels (Figure 3A,D). Interestingly, Major histocompatibility complex (MHC) II cell surface receptor (HLA-DR) was detected only in the native samples, ETN and EPN, as it was no longer expressed after decellularization, thus proving the low immunogenicity of the esophageal grafts (Figure 3C,F).

### 3.4. Quantitative Analysis of ECM Components

**Figure 4:**
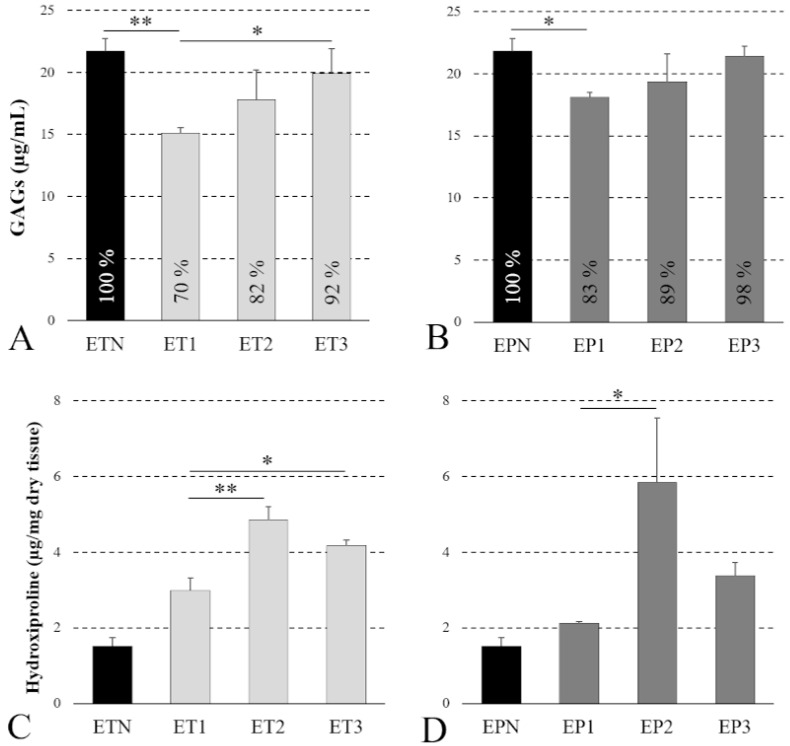
GAG and collagen preservation. Quantification of residual GAGs (**A**,**B**) and hydroxyproline (**C**,**D**) into esophageal tubules (ETs) and patches (EPs) before (ETN, EPN) and after decellularization with Protocols Nos. 1 (ET1, EP1), 2 (ET2, EP2) and 3 (ET3, EP3) (* *p* < 0.05; ** *p* < 0.01).

Starting from both native and decellularized specimens, the amount of GAGs and hydroxyproline was estimated via a biochemical assay to verify the preservation of these ECM components after detergent-enzymatic treatments (Figure 4).

Some differences among protocols were detected with these analyses, being GAGs significantly decreased in samples ET1 and EP1 compared with the native tissues, whereas they were found to be well preserved in scaffolds ET2, ET3, EP2 and EP3 (i.e., no significant differences compared with native samples) (Figure 4A,B). However, no massive depletion of GAGs was caused by Protocol No. 1, with samples ET1 and EP1 still retaining more than the 70–80% of native GAG content. Similarly, a better preservation of hydroxyproline was achieved with Protocols Nos. 2 and 3, rather than Protocol No.1. In particular, a significantly higher hydroxyproline amount was found in esophageal tubules ET2 and ET3 versus ET1 and in esophageal patches EP2 versus EP1 (Figure 4C,D).

### 3.5. Characterization of the ECM Collagen Component

**Figure 5:**
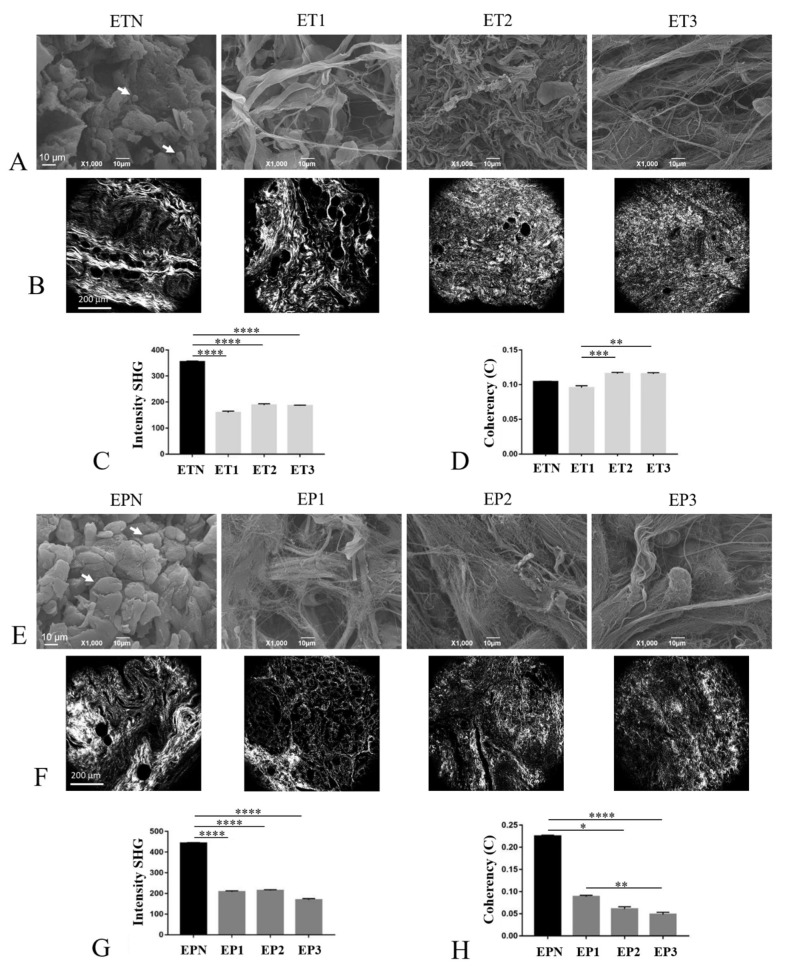
Characterization of collagen component. Collagen investigation in esophageal tubules (**A**–**D**) and patches (E-H) before (ETN, EPN) and after decellularization with Protocols Nos. 1 (ET1, EP1), 2 (ET2, EP2) and 3 (ET3, EP3). The collagen ultrastructure was analyzed using SEM, and the white arrows show the cells within the ETN and EPN scaffolds (**A**,**E**). SHG microscopy allowed us to localize specific collagen signals (**B**,**F**), measure signal intensity (**C**,**G**) and calculate coherency values for fiber orientation (**D**,**H**). Scale bars: 10 µm (**A**,**E**); 200 µm (**B**,**F**) (* *p* <0.05, ** *p* <0.01, *** *p* <0.001, **** *p* < 0.0001).

Collagens were among the most abundant and well-characterized components of the decellularized esophageal scaffolds. The SEM analysis confirmed ultrastructure maintenance after the decellularization of both tubules (Figure 5A and Figure S2A) and patches (Figure 5E and Figure S2B) in comparison with native samples. Cellular elements were detected in native esophagi (white arrows in Figure 5A,E and Figure S2) but not in decellularized matrices, with sample ET2 presenting crimped collagen fibers.

The investigation using SHG microscopy allowed us to characterize the composition of Collagen I and II, which give very strong signals [40] in both ET (Figure 5B) and EP (Figure 5F) samples versus the native tissues. The semi-quantitative analysis of collagen expression was coupled to the analysis of fiber spatial orientation, which is a crucial factor for the determination of the mechanical properties of a tissue. Specifically, the intensity of SHG is associated with the presence or absence of Collagen I and II, while the coherency parameter (C) permits to estimate the local orientation of the collagen fibers. The analysis demonstrated a significant decrease in SHG intensity in decellularized ET and EP samples compared with ETN and EPN, respectively (*p* < 0.0001) (Figure 5C,G). Furthermore, in esophageal tubules, collagen fibers showed to be orientated in every direction, with low values of C. No significant differences were detected between decellularized and native tubular samples, with both being characterized by an isotropic behavior (spherical shape for FFT; Figure 5D and Supplementary Figure S1A). Among decellularized ETs, the coherency values were similar for ET2 and ET3 samples, whereas a significant decrease in C parameters was detected for ET1 compared with ET2 (*p* < 0.001) and ET3 (*p* < 0.01). Considering esophageal patches, in EPN samples, collagen fibers tended to be predominately orientated in one direction, with an anisotropic behavior (ellipsoidal shape for FFT; Figure 5H and Figure S1B), while the decellularized samples were characterized by fibers orientated in every direction, with an isotropic behavior (spherical shape for FFT; Figure S1B). A statistically significant decrease in C values was detected in EP3 compared with EP1 (*p* < 0.01).

### 3.6. Proteomic Analysis of Decellularized Tissue Secretome

**Figure 6:**
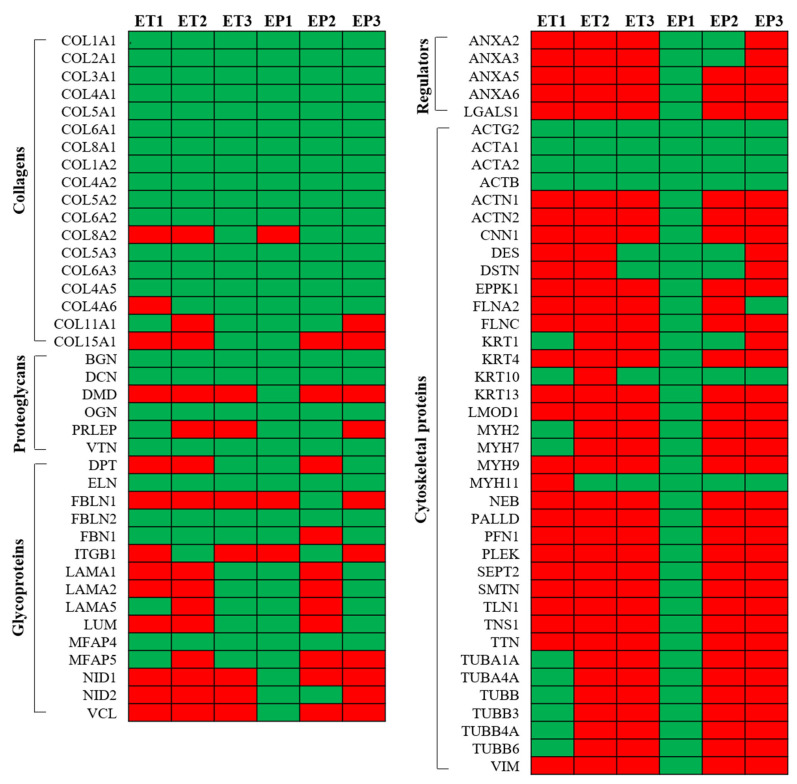
Proteomic study. Overview of the main detected proteins in esophageal tubules (ETs) and patches (EPs) decellularized with Protocols Nos. 1 (ET1, EP1), 2 (ET2, EP2) and 3 (ET3, EP3). (Green: positive protein expression; red: negative protein expression).

The preservation of tissue-specific proteins after decellularization was evaluated using mass spectrometry analysis. A total of 218 proteins were obtained after LC-MS/MS data processing, which were chosen and stratified in five main categories: collagens, proteoglycans, glycoproteins, cytoskeletal proteins and regulators. The total proteins detected within decellularized tubules and patches are presented in Figure 6, with the indication of positive (green) and negative (red) expression. This classification was chosen in order to highlight the impacts of the different decellularization protocols on ECM components having different solubility (collagens, proteoglycans and glycoproteins), on secreted proteins (regulators) and on possible contaminants (cytoskeletal proteins). The absolute value of proteins for each decellularized sample was calculated. Collagens resulted to be the most abundant proteins and included fibril-forming collagens (*COL1*, *COL2*, *COL3*, *COL5*, *COL11* and *COL15*), basal lamina collagens (*COL4*), cell–matrix bridging collagens (*COL6*) and non-fibrillar collagens (*COL8*). All detected collagens had similar distribution in all decellularized samples. The second class of main detected proteins was cytoskeletal proteins (*ACTG2*, *ACTA1*, *ACTA2*, *ACTB*, *ACTN1*, *ACTN2*, *CNN1*, *DES*, *DSTN*, *EPPK1*, *FLNA2*, *FLNC*, *KRT1*, *KRT4*, *KRT10*, *KRT13*, *LMOD1*, *MYH2*, *MYH7*, *MYH9*, *MYH11*, *NEB*, *PALLD*, *PFN1*, *PLEK*, *SEPT2*, *SMTN*, *TLN1*, *TNS1*, *TTN*, *TUBA1A*, *TUBA4A*, *TUBB*, *TUBB3*, *TUBB4A*, *TUBB6* and *VIM*), which were similarly maintained in ET1, ET3, EP2 and EP3. In parallel, ET2 and EP1 samples showed to retain, respectively, the lowest (*n* = 4) and the highest (*n* = 37) numbers of cytoskeletal proteins after decellularization treatment. Similar to collagens, all the decellularization protocols seemed to comparably preserve proteoglycans (*BGN*, *DCN*, *DMD*, *OGN*, *PRLEP* and *VTN*). Regarding glycoprotein (*DPT*, *ELN*, *FBLN1*, *FBLN2*, *FBN1*, *ITGB1*, *LAMA1*, *LAMA2*, *LAMA5*, *LUM*, *MFPA4*, *MFPA5*, *NID1*, *NID2* and *VCL*) stratification, higher numbers were observed in EP1, ET3 and EP3 samples compared with ET1, ET2 and EP2 scaffolds. Lastly, after decellularization, regulators (ANXA2, ANXA3, ANXA5, ANXA6 and LGALS1) were found to be still retained in EP1 and EP2 samples, while they were not detected in the remaining acellular scaffolds.

### 3.7. Mechanical Behavior

**Figure 7:**
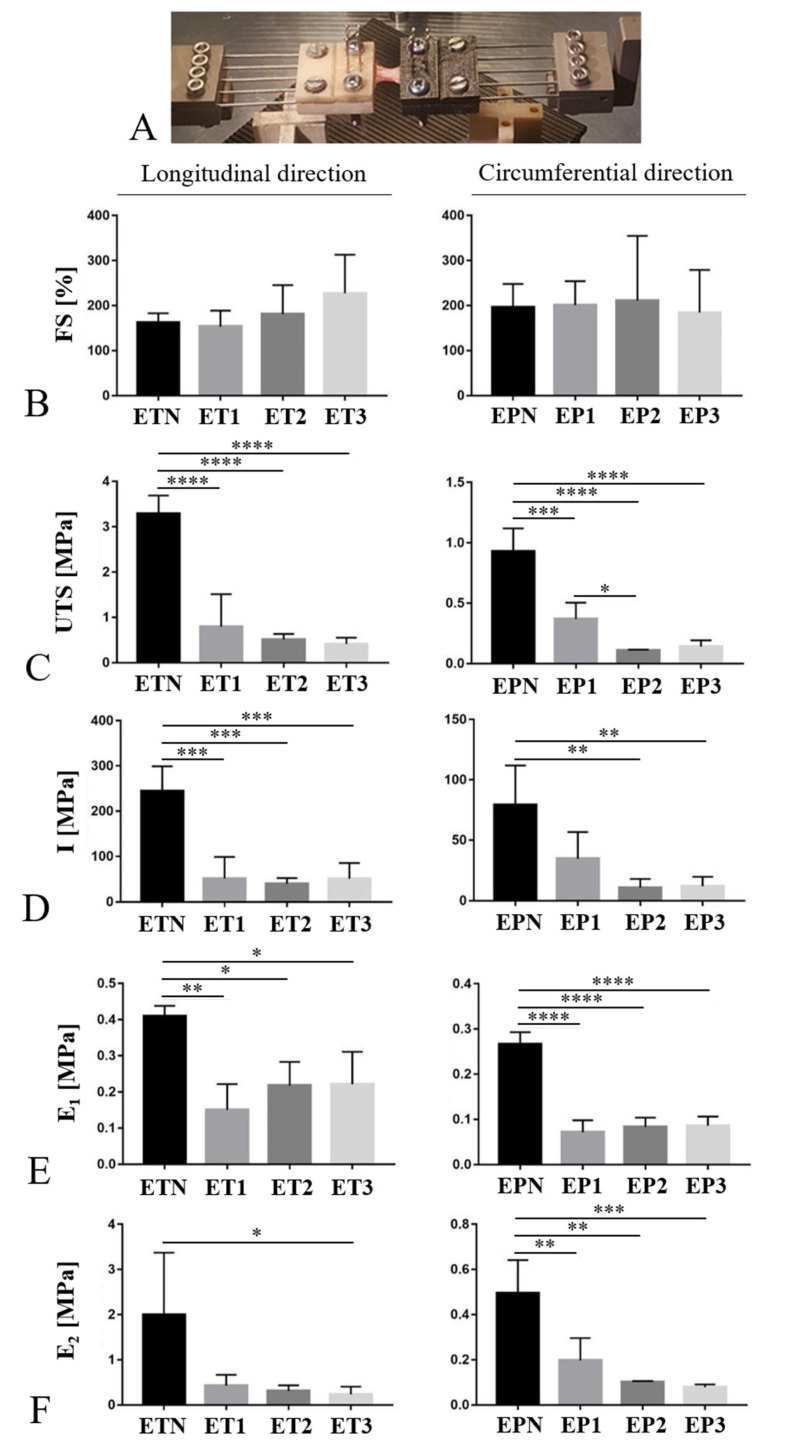
Biomechanics of decellularized esophagi. Mechanical properties of esophageal patches before (EPN) and after decellularization with Protocols Nos. 1 (EP1), 2 (EP2) and 3 (EP3). The tensile force was applied using a custom-made apparatus (**A**) along both longitudinal and circumferential directions to measure Failure Strain (FS) (**B**), Ultimate Tensile Strength (UTS) (**C**) and toughness (I) (**D**). Young’s modules E_1_ (**E**) and E_2_ (**F**) were also calculated as the slope of the stress–strain curves at a deformation of 1–10% and 80–90%, respectively (* *p* <0.05, ** *p* <0.01, *** *p* <0.001, **** *p* < 0.0001).

The gross appearance of esophageal samples during the mechanical test is shown in Figure 7A. After measuring the FS, which represents the maximum elongation of the sample, no differences were found between native and decellularized tissues for neither the longitudinal nor circumferential direction (Figure 7B). Regarding the maximum resistance of the sample before rupture (UTS), a significant decrease was registered in decellularized esophagi compared with the native tissues both in the longitudinal and circumferential directions of analysis (Figure 7C). Furthermore, a significant UTS reduction was observed for EP2 as compared with EP1 tested along the circumferential direction (Figure 7C). Tissue toughness (I) resulted to be significantly affected by all the three decellularization methods in samples elongated in the longitudinal direction (Figure 7D). Conversely, in the experimental group that was circumferentially elongated, a significant toughness reduction was observed for the EP2 and EP3 samples in comparison with EPN (Figure 7D). Finally, a clear reduction in Young’s modules E_1_ and E_2_ was also registered after decellularization with all the three methods (Figure 7E,F), although no significant differences were calculated between the EP1 or EP2 sample and the native tissue when samples were elongated in the longitudinal direction.

### 3.8. Cytotoxicity Study

**Figure 8:**
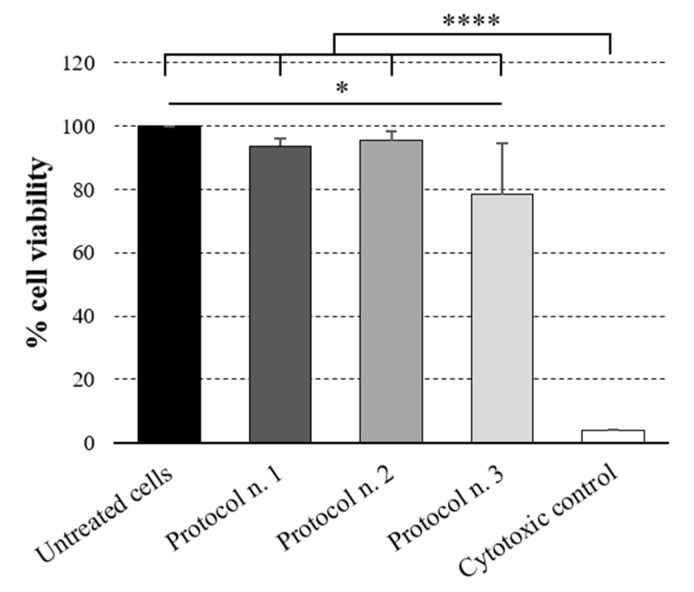
Cytocompatibility study. Cytotoxicity test on HM1-SV40 cell line incubated for 72 h with culture media conditioned with esophageal matrices decellularized through Protocols Nos. 1, 2 and 3. For each experimental group, the percentage of cell viability after the incubation period was defined in comparison with the untreated cell cultures (100% viability) (* *p* < 0.05; **** *p* < 0.0001).

After the 72 h exposure period to culture media conditioned with decellularized esophageal samples, HM1-SV40 cells appeared to be viable and proliferating, reaching about 90% confluence on the growth surface. The MTT assay revealed that, compared with untreated cultures, cells preserved 93.7%, 95.4% and 78.3% viability when treated with medium conditioned with esophageal matrices decellularized through Protocols No. 1, No. 2 and No. 3, respectively (Figure 8). Significant differences were detected between the cytotoxic control and all other samples (*p* < 0.0001), confirming that cell cultures responded appropriately in comparison with the positive control. A significant decrease in cell viability (*p* < 0.05) was found to be caused by medium conditioned with esophageal tissues treated with Tergitol^TM^ with respect to the untreated control. However, more than 70% cell viability was still preserved, in line with the set threshold value which defines a non-toxic device [41] (Figure 8).

## 4. Discussion

Acellular scaffolds prepared via decellularization methods are a promising tool in TE to drive the development of off-the-shelf tissue replacements for future-generation therapies of organ repair/reconstruction. These scaffolds are based on the preservation of tissue ECM, which offers the advantage of faithfully recreating the three-dimensional macro- and microarchitecture of the target organ [42,43,44], also guiding cell adhesion, migration, proliferation and organization in vivo [1].

The functional reconstruction of a defective esophagus still represents a challenge for modern gastroenterological surgery, with TE being a promising approach to face currently unmet clinical needs. Specifically, biological scaffolds based on acellular matrices seem to be the most suitable option to obtain biocompatible tissue substitutes for full-thickness esophageal repair [17]. Esophageal acellular matrices have been obtained by means of different processing methodologies and from a diversity of species, including rat [5,45,46,47,48,49,50,51], rabbit [52,53,54], sheep [55], goat [56] and pig [10,18,57,58,59,60,61,62,63,64,65,66]. To the best of the authors’ knowledge, human esophagus has not been investigated so far to prepare decellularized biocompatible matrices for esophageal reconstruction. This represents a significant research gap when considering that human acellular matrix grafts—derived from the same species of the recipient patient—may ensure better biocompatibility, biomolecular composition, biomechanical properties and bio-integration abilities than their animal counterparts, also minimizing risks of infection [24,44]. Based on that, the present work compared three different protocols for human esophagus decellularization and performed a detailed characterization of the resulting acellular matrices. We decided to work with esophageal scaffolds with two different gross morphologies, i.e., patches and tubular segments, in order to produce implantable devices that can serve, respectively, for the repair of wall defects or for the circumferential replacement of esophageal tracts. Esophageal samples were harvested from donated bodies who were normally frozen to prevent cadaver tissue deterioration (i.e., morphological alterations and damage due to drying, autolysis and putrefaction). The freezing/thawing of tissues is considered a physical decellularization method, which results in cell lysis without significantly disrupting the ultrastructure of the original specimen [39]. Thus, using frozen esophageal samples is known to help with the process of cell/nuclear DNA removal. In this work, the decellularization methods consisted of detergent-enzymatic treatments based on the use of different detergents, namely, sodium deoxycholate (SDC) (Protocol No. 1), sodium dodecyl sulphate (SDS) (Protocol No. 2) and Tergitol^TM^ (Protocol No. 3). Regarding enzymes, DNase I was used in Protocol No. 1 to completely remove nuclear genetic material after cell lysis [39], whereas Protocols No. 2 and No. 3 took advantage of trypsin, which digests membrane proteins, leading to cell death, and—in combination with EDTA—also contributes to breaking the cell adhesion to the matrix [67]. Given its specific proteolytic activity, trypsin was applied by reducing the processing time and together with a chelating agent, so that undesired degradation effects on ECM components (i.e., collagen, elastin, GAGs) were prevented [68]. Regarding enzymes for decellularization, being nucleases very expensive reagents, the possibility to effectively produce acellular esophageal matrices without using them may represent an important advantage in terms of procedure costs. 

SDC- and DNase-based protocols have already been reported to be effective for the decellularization of rat [5], rabbit [53] and pig [10] esophagi, whereas a trypsin-based method including Triton X-100 detergent has allowed skeletal muscle acellular matrix to be successfully produced [23]. However, since Triton X-100 cannot be used for the preparation of implantable materials anymore due to cytotoxicity concerns, two alternative detergents were tested as possible substitutes. Esophagus decellularization aimed to eliminate all cellular and nuclear antigens while minimizing any adverse effects on the structural composition, organization and integrity of the remaining ECM. The efficacy of all the tested protocols was substantiated via H&E, showing no visible nuclear material in none of the decellularized samples. In parallel, the DNA quantification analysis proved that SDC and DNase I more successfully removed immunogenic nucleic acids, although the other two methods also showed to lower the DNA content below the maximum limit for decellularized scaffolds. The non-immunogenicity of esophageal tubules and patches after decellularization was also corroborated by the immunolocalization of HLA-DR, which is a major histocompatibility complex (MHC) II cell surface receptor associated with poor graft integration and rejection events [69]. Differently from native esophagus, which showed positive conservation, acellular grafts completely lost this antigen, suggesting high biocompatibility rates in case of in vivo implant. The validation of decellularization protocols implies the need to prove that the native-tissue microarchitecture has been preserved. Indeed, eliminating cellular/nuclear antigens and preserving ECM composition is a fine balance, which needs to be considered for the development of biocompatible scaffolds with ideal biomolecular and mechanical cues. Herein, a good maintenance of elastic and collagen fibers in all decellularized esophagi was demonstrated via histological investigations, whereas the muscular component was removed by employing all the processes, with slightly more preservation in samples treated using Protocol No. 1. Importantly, histological sections confirmed that the vascular elements were retained after detergent-enzymatic treatments, which is necessary for tissue nutrition and material exchange in the perspective of in vivo implants [70]. Within the ECM, the role of GAGs is to bind growth factors and cytokines, as well as to retain water molecules in tissue. In this work, the biochemical assay for GAG quantification revealed that a better preservation of this component was assured using Protocols Nos. 2 and 3, rather than Protocol No. 1, in line with other studies reporting the depletion of GAGs after esophagus decellularization using an SDC-based protocol [62]. However, only a moderate decrease in the GAG amount was registered in comparison with previous studies, suggesting that ET1 and EP1 samples can still take advantage of the functional role of GAGs in case of scaffold repopulation. In parallel, these results are in contrast with those of several works describing the extraction of GAG following trypsin- or SDS-based procedures [71]. Together with GAGs, the collagen content was determined via hydroxyproline biochemical quantification, since hydroxyproline is present almost exclusively in collagen. Similarly, in this case, higher amounts of hydroxyproline were detected after decellularization with Protocols No. 2 and 3, although all acellular scaffolds showed an increase in hydroxyproline content after decellularization, which could be explained by the fact that cells and possibly some proteoglycans no longer contributed to tissue weight [56]. Specifically, the detected hydroxyproline content was normalized to the specific sample weight; in native tissue, for the same weight, there are both cellular components and structural proteins, while in decellularized ones there are no cellular components. So, the apparent increase in hydroxyproline was probably due to the loss of cells and soluble proteins in decellularized tissues in comparison with the native counterparts [72]. 

Remarkably, the preservation of ECM components after treatment with trypsin-based methods can be attributed to the limited time of incubation in the proteolytic enzyme solution. Besides hydroxyproline quantification, Collagen IV, together with laminin, was still localized within the esophageal matrices after decellularization, representing fundamental structural components that endow the acellular scaffold with mechanical strength and affinity for cells [73]. The successful outcome of decellularized tissues upon implantation is ascribed to the molecular signals provided by the remaining ECM components, which can crosstalk with cells in vivo as they repopulate the graft. The proteomic signature of esophageal acellular matrix also demonstrates the retention of structural collagens, besides basement membrane and matrix–cell interaction proteins. In accordance with a previous characterization of pig esophageal ECM [61], we observed that acellular tubules and patches were mainly made of fibrillar collagens, including types I, III and V, which define the physical characteristics of the tissue. Glycoproteins, which constitute elastic fibers (elastin, fibrillin, microfibrillar-associated proteins), seemed to be well maintained through all protocols, while those forming the basement membrane (laminin, fibulin, nidogen) were more preserved using Protocols No. 1 and No. 3. Proteoglycans, which play a role in matrix assembly (biglycan, decorin) and structural regulation (osteoglycin), as well as cell adhesion (vitronectin), were found not to be depleted due to decellularization. Cytoskeleton proteins are known to be fundamental for the structural/functional organization of the cell, so their persistence in acellular matrices may be a sign of incomplete cell removal. Comparing the three decellularization methods, this class of proteins was mainly detected within esophageal tissues treated with Protocol No. 1, suggesting the presence of cell remnants. On the other hand, Protocol No. 1, followed by Protocol No. 2, ensured a better preservation of some regulator factors that are implicated in the modulation of cell–matrix interactions (annexin, galectin).

Since the cell behavior in the ECM (i.e., adhesion, migration, proliferation gene expression) is believed to be influenced not only by the tissue-specific proteome but also by the surface morphology of the scaffolds [74,75], an in-depth ultrastructural investigation of native and acellular esophagi was performed using SEM. The superficial collagen structure after decellularization consisted of fine, loosely arranged, undulating collagen bundles that were separated from each other by irregular spaces, while intact cells were no more visible. As a further characterization of the collagenous component within native and acellular esophageal specimens, the SHG signal collected from collagen allowed us to obtain a semi-quantitative evaluation of its fibrillar structure [34]. The current literature about esophagus decellularization has never reported this type of analysis on the acellular matrix; still, it was shown to provide useful insights in decellularization–recellularization studies to discriminate between the collagen/elastin of the scaffolds and the original cells or the recellularizing tissue [76]. The SHG imaging of decellularized versus native tissues was previously found to detect a significant decrease in signal intensity from collagen fibers of native samples after decellularization. This signal reduction was associated with structural damage to collagen due to detergent-enzymatic treatment [77,78]. This study appears to be in line with these data, highlighting that all the tested protocols caused a decrease in collagen signal intensity compared with the untreated esophagi. On the other hand, collagen fiber orientation seemed to be less affected by the decellularization process in tubular samples rather than patches, suggesting that the gross morphology of the scaffold may influence tissue response to treatment. However, the alterations in collagen fiber orientation following decellularization treatment have already been reported in the literature for porcine corneas [79]. Moreover, trypsin/EDTA treatment has been found to disorganize the collagen fiber orientation in decellularized bovine bone [80].

After structural characterization, the biomechanical investigation of acellular scaffolds is of pivotal importance to establish the correct preservation of tissue functional integrity. In this study, all the tested decellularization methods led to a decrease in mechanical resistance and toughness in comparison with the native counterpart. Collected evidence on the biomechanical properties of esophageal tissue after decellularization appears to be controversial, since some authors have reported a reduction in scaffold stiffness [18,47], while others have confirmed the preservation of adequate mechanical performance [54,56]. In this study, the loss of tissue mechanical performance may have been ascribed to the fact that the esophageal samples were frozen before processing. In fact, controversial evidence has been reported in the literature about the loss of sample stiffness after freezing/thawing, with several studies detecting little or no mechanical changes in frozen versus fresh ligaments, tendons, menisci, and arterial and ocular tissues [81]. Based on that, the residual biomechanical properties of the acellular esophageal scaffolds obtained in this work are to be challenged with scaffold repopulation tests.

Another consideration about the effect of the different decellularization methods on tissue mechanical properties regards the use of trypsin for the enzymatic dissociation of cell–matrix interactions. When using trypsin, treatment duration is of key importance to minimize adverse effects on ECM structure and mechanical behavior, as it has been reported in the literature that a prolonged incubation up to 15–24 h affects matrix stiffness and biomechanics [82,83]. In this study, samples were maintained in trypsin for a limited time (1 h), so it is likely that it helped not to affect Young’s modulus more than treatments based on detergent (SDC) alone.

Besides their mechanical, physical and chemical properties, medical devices such as engineered allografts must undergo rigorous testing to assess their biocompatibility and bio-safety properties before they can be implanted in vivo [84]. In this regard, in vitro cytotoxicity tests represent the primary crucial step to predict the behavior of an implantable scaffold for possible clinical application. Herein, cytocompatibility tests performed on the HM1-SV40 cell line supported the atoxicity of all the acellular esophageal matrices, with better results having been obtained with decellularization Protocols No. 1 and No. 2. These encouraging results need to be corroborated with in vivo studies assessing the biocompatibility of the decellularized esophagi after the subcutaneous implant into rat/mouse models.

## 5. Conclusions

The failure of esophageal structure and function often requires complex reconstructive surgery with discussible long-term outcomes; hence, new and better therapeutic approaches strongly rely on advances in tissue engineering. In this work, non-cytotoxic esophageal allografts were obtained using three different protocols that gently removed cells and genetic material to preserve the ECM structure and the micro- and macro-architecture of the native tissue, also retaining crucial biological cues, as detected via a proteomic study.

The three decellularization protocols provided different outcomes in terms of (a) tissue manipulability (more affected by Protocol No. 3), (b) DNA removal (better achieved via Protocol No. 1), (c) GAG and hydroxyproline preservation (better assured by Protocols Nos. 2 and 3) and (d) collagen fiber orientation (less affected by Protocols Nos. 2 and 3 for ET samples and by Protocol No. 1 for EP specimens). The proteomic signature of acellular esophagi also showed some differential expression among experimental groups, suggesting that different biological signals can be provided by the decellularized matrices according to the protocol used. Overall, the in vitro results did not show the clear superiority of one protocol over the others, suggesting that the proper decellularization method may be chosen according to the different therapeutic and regenerative demands, so that personalized scaffolds may be produced to better satisfy patients’ needs. Most importantly, further studies investigating in vivo matrix biocompatibility, scaffold repopulation and orthotopic implant into esophageal damage/pathology models should corroborate the quality of the different acellular esophageal grafts, eventually underlying more distinct differences among the methods.

## Figures and Tables

**Table 1:** Decellularization methods. Detergent-enzymatic protocols tested on human esophageal tubules and patches. Agitation speed was 100 oscillations/minute.

Protocol No.	1	2	3
**Method**	dH_2_O (overnight at 4 °C) 4% SDC (4 h at RT, under agitation) 2000 kU DNase I in 1 M NaCl (3 h at RT, under agitation) dH_2_O (overnight at 4 °C) After the last cycle: PBS 1X + 3% Pen/strep (72 h at 4 °C, under agitation) Peracetic acid 0.1 M (1 h at RT, under agitation)	dH_2_O (24 h at 4 °C) 0.05% Trypsin + 0.02% EDTA in PBS (1 h at 37 °C) 0.002% SDS + 0.8% NH_4_OH in PBS (72 h at 4 °C, under agitation) dH_2_O (72 h at 4 °C)	dH_2_O (24 h at 4 °C) 0.05% Trypsin + 0.02% EDTA in PBS (1 h at 37 °C) 2% Tergitol^TM^ + 0.8% NH_4_OH in PBS (72 h at 4 °C, under agitation) dH_2_O (72 h at 4 °C)

**Abbreviations:** dH_2_O, deionized water; DNase I, Deoxyribonuclease I; EDTA, Ethylenediaminetetraacetic acid; NaCl, sodium chloride; NH_4_OH, ammonium hydroxide; PBS, phosphate-buffered saline; Pen/strep, penicillin/streptomycin; SDC, sodium deoxycholate; SDS: Sodium dodecyl sulphate.

**Table 2:** Quantitative measures of sample appearances. Weight, external diameter and length of esophageal tubules (ETs) and patches (EPs) before (ETN, EPN) and after decellularization with Protocols Nos. 1 (ET1, EP1), 2 (ET2, EP2) and 3 (ET3, EP3).

Sample	Weight (g)	Width (cm)	Length (cm)
ETN	4.27 ± 0.12	1.8 ± 0.01	2.1 ± 0.14
ET1	4.73 ± 0.24	2.4 ± 0.05	2.1 ± 0.05
ET2	4.81 ± 0.11	1.9 ± 0.01	2.4 ± 0.14
ET3	4.95 ± 0.09	2.0 ± 0.03	2.0 ± 0.07
EPN	2.06 ± 0.09	2.0 ± 0.04	2.4 ± 0.06
EP1	2.45 ± 0.52	1.9 ± 0.01	2.9 ± 0.02
EP2	2.53 ± 0.34	2.1 ± 0.12	4.5 ± 0.10
EP3	2.69 ± 0.20	2.3 ± 0.12	4.1 ± 0.23

## Supplementary Materials

The following supporting information can be downloaded at: https://www.mdpi.com/article/10.3390/cells11192945/s1, Figure S1: Collagen fiber orientation; Figure S2: Surface ultrastructure of native and decellularized esophagi.

## Abbreviations

ACTA1	Actin, Alpha, Skeletal Muscle 1
ACTA2	Actin, Alpha-2, Smooth Muscle, Aorta
ACTB	Actin, Beta
ACTG2	Actin, Gamma-2, Smooth Muscle, Enteric
ACTN1	Actinin, Alpha-1
ACTN2	Actinin, Alpha-2
ANXA2	Annexin A2
ANXA3	Annexin A3
ANXA5	Annexin A5
ANXA6	Annexin A6
BGN	Biglycan
CNN1	Calponin 1
COL1A1	Collagen, Type I, Alpha-1
COL1A2	Collagen, Type I, Alpha-2
COL2A1	Collagen, Type II, Alpha-1
COL3A1	Collagen, Type III, Alpha-1
COL4A1	Collagen, Type IV, Alpha-1
COL4A2	Collagen, Type IV, Alpha-2
COL4A5	Collagen, Type IV, Alpha-5
COL4A2	Collagen, Type IV, Alpha-6
COL5A1	Collagen, Type V, Alpha-1
COL5A2	Collagen, Type V, Alpha-2
COL5A3	Collagen, Type V, Alpha-3
COL6A1	Collagen, Type VI, Alpha-1
COL6A2	Collagen, Type VI, Alpha-2
COL6A3	Collagen, Type VI, Alpha-3
COL8A1	Collagen, Type VIII, Alpha-1
COL8A2	Collagen, Type VIII, Alpha-2
COL11A1	Collagen, Type XI, Alpha-1
COL15A1	Collagen, Type XV, Alpha-1
DCN	Decorin
DES	Desmin
DPT	Dermatopontin
DSTN	Destrin
DMD	Dystrophin
ELN	Elastin
EPPK1	Epiplakin 1
FBN1	Fibrillin 1
FBLN1	Fibulin 1
FBLN2	Fibulin 2
FLNA2	Filamin
FLNC	Filamin C
ITGB1	Integrin, Beta-1
KRT1	Keratin 1, Type II
KRT10	Keratin 10, Type I
KRT13	Keratin 13, Type I
KRT4	Keratin 4, Type II
LAMA1	Laminin, Alpha-1
LAMA2	Laminin, Alpha-2
LAMA5	Laminin, Alpha-5
LGALS1	Lectin, Galactoside-Binding, Soluble, 1
LMOD1	Leiomodin 1
LUM	Lumican
MFAP4	Microfibrillar-Associated Protein 4
MFAP5	Microfibrillar-Associated Protein 5
MYH11	Myosin, Heavy Chain 11, Smooth Muscle
MYH2	Myosin, Heavy Chain 2, Skeletal Muscle, Adult
MYH7	Myosin, Heavy Chain 7, Cardiac Muscle, Beta
MYH9	Myosin, Heavy Chain 9, Nonmuscle
NEB	Nebulin
NID1	Nidogen 1
NID2	Nidogen 2
OGN	Osteoglycin
PALLD	Palladin, Cytoskeletal-Associated Protein
PFN1	Profilin 1
PLEK	Pleckstrin
PRLEP	Prolargin
SEPT2	Septin 2
SMTN	Smoothelin
TLN1	Talin 1
TNS1	Tensin 1
TTN	Titin
TUBA1A	Tubulin, Alpha-1a
TUBA4A	Tubulin, Alpha-4a
TUBB	Tubulin, Beta
TUBB3	Tubulin, Beta-3
TUBB4A	Tubulin, Beta-4a
TUBB6	Tubulin, Beta-6
VCL	Vinculin
VIM	Vimentin
VTN	Vitronectin
