# Supplementary material for: Bio-Engineered Scaffolds Derived from Decellularized Human Esophagus for Functional Organ Reconstruction
Source: Cells. 2022 Sep 20;11(19):2945. doi: 10.3390/cells11192945 (PMC9563623; doi:10.3390/cells11192945)
Supplement: Supplementary file 1 [file cells-11-02945-s001.zip › cells-1896393-supplementary.pdf]

cells

MDPI

Supplementary Material

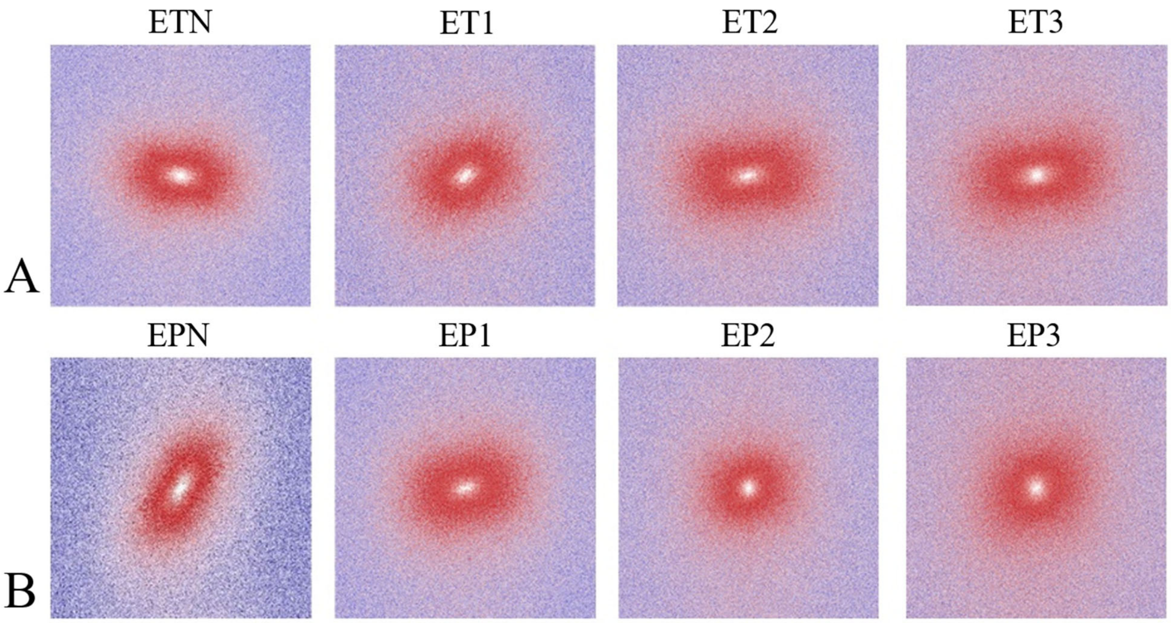

Figure S1. Collagen fiber orientation. Fast Fourier Transform (FFT) analysis on esophageal tubules (ETs) (**A**) and patches (EPs) (**B**) before (native: ETN, EPN) and after decellularization with Protocols Nos. 1 (ET1, EP1), 2 (ET2, EP2) and 3 (ET3, EP3).

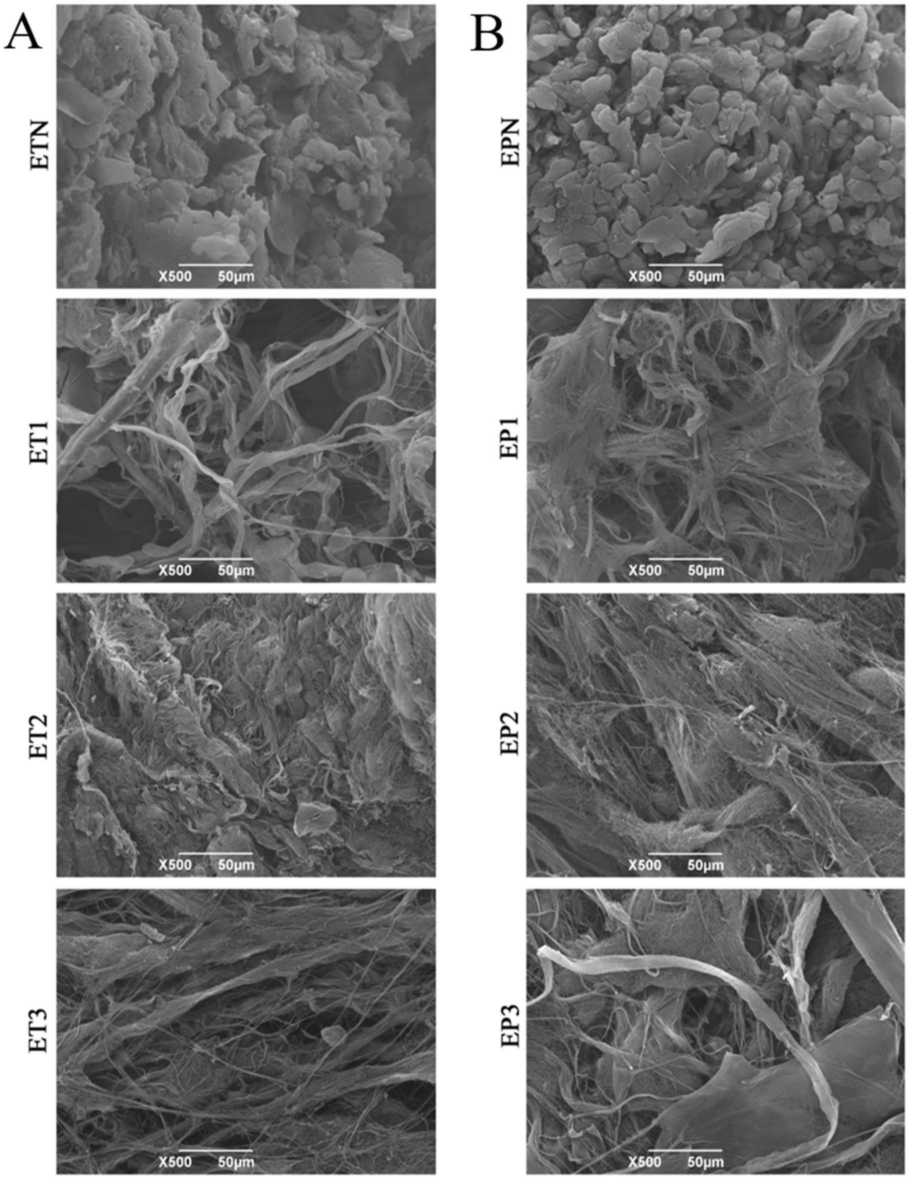

Figure S2. Surface ultrastructure of native and decellularized esophagi. SEM micrographs showing the presence of cellular elements on the surface of native esophageal tubules (ETN) and patches (EPN). In esophageal tubules (ETs) (**A**) and patches (EPs) (**B**) decellularized with Protocols Nos. 1 (ET1, EP1), 2 (ET2, EP2) and 3 (ET3, EP3), the collagen fiber arrangement can be observed. Scale bar: 50 µm.
